# A Game-Theoretical Network Formation Model for *C. elegans* Neural Network

**DOI:** 10.3389/fncom.2019.00045

**Published:** 2019-07-09

**Authors:** Mohamad Khajezade, Sama Goliaei, Hadi Veisi

**Affiliations:** ^1^Laboratory for Computational Biology and Bioinformatics, Department of New Sciences and Technologies, University of Tehran, Tehran, Iran; ^2^The Data and Signal Processing Laboratory, Department of New Sciences and Technologies, University of Tehran, Tehran, Iran

**Keywords:** complex network analysis, game theory, network formation models, *C. elegans* frontal neural network, *C. elegans* neural network, computational neuroscience

## Abstract

Studying and understanding human brain structures and functions have become one of the most challenging issues in neuroscience today. However, the mammalian nervous system is made up of hundreds of millions of neurons and billions of synapses. This complexity made it impossible to reconstruct such a huge nervous system in the laboratory. So, most researchers focus on *C. elegans* neural network. The *C. elegans* neural network is the only biological neural network that is fully mapped. This nervous system is the simplest neural network that exists. However, many fundamental behaviors like movement emerge from this basic network. These features made *C. elegans* a convenient case to study the nervous systems. Many studies try to propose a network formation model for *C. elegans* neural network. However, these studies could not meet all characteristics of *C. elegans* neural network, such as significant factors that play a role in the formation of *C. elegans* neural network. Thus, new models are needed to be proposed in order to explain all aspects of *C. elegans* neural network. In this paper, a new model based on game theory is proposed in order to understand the factors affecting the formation of nervous systems, which meet the *C. elegans* frontal neural network characteristics. In this model, neurons are considered to be agents. The strategy for each neuron includes either making or removing links to other neurons. After choosing the basic network, the utility function is built using structural and functional factors. In order to find the coefficients for each of these factors, linear programming is used. Finally, the output network is compared with *C. elegans* frontal neural network and previous models. The results implicate that the game-theoretical model proposed in this paper can better predict the influencing factors in the formation of *C. elegans* neural network compared to previous models.

## 1. Introduction

One of the major goals in neuroscience is studying and understanding human brain functions and structures. Various methods and tools are used to find a way to map the human brain. However, the mammalian brain has hundreds of thousands of neurons connecting through billions of synapses (Huttenlocher, 1984). For instance, as reported in The European Union human brain project official web site, The human brain is consist of 86 billion neurons and each neuron has 7,000 connections on average. This complexity makes it impossible to models the human brain using current computers (European-Union, 2017). It is clear that finding the signal between each neuron with other neurons in the human brain in the laboratory is nearly impossible and time-consuming. So, this complexity makes it impossible to study such huge systems by biological reconstruction in the laboratory.

One way to deal with this complexity is by describing the nervous system in a computational way. Computational neuroscience is the science of investigating brain and the way the nervous system generates certain behaviors in a computational manner (Feng, 2003). The complex network analysis is a part of computational neuroscience that is used to study structures and features of the nervous system.

The nervous system can be modeled as a complex network of neurons, the population of neurons, and the brain regions as nodes (Sporns, 2012). In this network, the chemical and electrical synapses and fiber tracks can be considered as connections or links between neurons and population of neurons as well as brain regions(Kaiser and Varier, 2011). In the neural network, links can be sometimes something other than biological links. They may connect regions or neurons that show similar patterns of activity. The networks formed by biological links are called structural networks while others are called functional networks. Studies show that there are definitive and meaningful relations between structural and functional networks (Stam et al., 2016; Reimann et al., 2017). Thus, studying structural networks can shade light into functional properties.

While some studies that use computational models and complex network analysis are conducted on the human brain, it is nearly impossible to simulate human brain structure due to complexity (Sporns et al., 2005; Elliott et al., 2017). Most of these studies focus on human brain functional networks and they usually fail to regenerate structural properties. So, a simpler nervous systems should be a better start to model and simulate structural properties of biological neural networks. *C. elegans* neuronal network is the only neuronal network that is reported to be nearly fully mapped (Jarrell et al., 2012). Although the *C. elegans* neural network is simple, yet basic behaviors, such as head movements, forward and backward locomotion, and turns emerge from this network (Chalfie et al., 1985). So, *C. elegans* neuronal network is one of the most favorite networks for neuroscientists who want to study a real neuronal network. The adult hermaphrodite worm has 302 neurons connecting through ~6,400 chemical synapses, 900 gap junctions, and 1,500 neuromuscular junctions (Jarrell et al., 2012).

Understanding how these neural networks evolved and why they are in the shape they are, can lead to a better understanding of the structure of nervous systems. In this paper, a new network formation model is proposed to investigate factors affecting neuronal networks development and secrets behind neuronal networks structures. The candidate factors investigated in this work includes physical distance between neurons, the difference between neurons' birth time, closeness centrality of neurons, betweenness centrality of neurons, neurons' page rand, and average shortest path between neurons.

Needless to say, there are other functional factors that can contribute to the formation of neural networks. For instance, recent studies show the importance of stochastic resonance (SR) in neural networks for information processing. These studies suggest that the brain works in a noisy environment. There is plenty of evidence indicating that neuronal noise might facilitate signal processing in neural networks through an SR behavior (Guo et al., 2017, 2018). Another study discusses that the structural heterogeneity of synaptic input connectivity in neural networks can describe the neuronal avalanches in the brain. Therefore, the structural heterogeneity can have an important role in the formation of the neural network (Wu et al., 2019).

The model provided in this paper is a strategic (game-theoretical) network formation model based on pairwise stability and is built up based on the *C. elegans* frontal neuronal network. The model is compared with previously proposed models for *C. elegans*. The remainder of the paper organized as follows: In the first section, the related literature is reviewed in order to let the reader follow up work, in the second section the methodology that is used to implement the model is described, finally in the discussion section, the model is evaluated and compared with previous studies.

## 2. Related Works

Coelenterates like Cnidaria were the first species to have the neuronal network (Spencer and Satterlie, 1980). Their neural network was a two-dimensional regular or lattice network (Watson and Augustine, 1982). It means that their neural network formed a regular network (Bergström and Nevanlinna, 1972). In regular networks, neighbors are well-connected and there is no link between the nodes in long distance (Jerauld et al., 1984). This type of network still can be seen in two-dimensional structures of the neuronal network, such as the retina, cortical, and sub-cortical layered structures(Bassett and Bullmore, 2017). However, regular networks fail to describe more complex neuronal networks when neural network wiring is a combination of genetic information, stochastic processes, and learning mechanisms (Walters and Byrne, 1983).

Some studies have proposed random networks like Erdos-Renye, and random scale-free networks to simulate, model or analysis of biological neural networks, such as the Macaque cortical connectome or the *C. elegans* frontal ganglia connectome (Prettejohn et al., 2011; Cannistraci et al., 2013).

In order to find the factor affecting network formation, Itzhack and Louzoun proposed a random model based on the distances between neurons (Itzhack and Louzoun, 2010). The model is a random network based on the Euclidean distance between each neuron. This random network is then compared against the *C. elegans* neural network. While the average shortest path between this model and the real network are similar, there is a huge difference between their clustering coefficients.

The model proposed by Itzhack and Louzoun can describe some characteristics of *C. elegans* neural network. However, sometimes neurons in long distances form synaptic links. To find the reason for the formation of links between neurons in long distance, Kaiser et al. have investigated the role of birth time in the formation of neurons and have demonstrated the effect of birth time in the formation of neuronal networks (Varier and Kaiser, 2011).

Although these models can describe some characteristics of neuronal networks, they failed to capture all the factors that affect neuronal network formation processes. These models take one or two structural or functional factors into account, individually. In the formation of neural networks, both structural and functional factors have major roles, and network formation models should take all these types of factors into account. In this paper, a game-theoretical network formation model for *C. elegans* frontal neural network is proposed to include both structural and functional factors in the formation of neural networks.

## 3. Methods

In this section, the new game-theoretic network formation model for the *C. elegans* frontal neuronal network is described. To model the network strategically, two aspects are important to consider. First, the benefit and the cost of the network should be identified. Second, agents' incentives need to be translated into the network benefit. In other words, the right strategies for agents should be chosen.

### 3.1. Strategic Network Formation Models

There are two different views on strategic network formation. One might form the links based on the nodes' incentive, benefits they gain or cost they pay through making connections with other nodes in the network. The second view takes the whole network into account and decides based on benefit and cost for the network (Jackson, 2005). Since neurons are added to the network one at the time, In this paper, the first view is used to form the model. In the proposed model, neurons assumed as agents. let *N* = {*n*_1_, *n*_2_, …, *n*_131_} be the set of agents as *n*_*i*_ demonstrate the *i*th neuron, and let *S* = {*L, D*} be the set of strategies for each neuron, where *S*_*i, j*_ = *L* shows the inclination of neuron *i*th to form the link with neuron *j*th and *S*_*i, j*_ = *D* shows otherwise.

Next, the Utility function should be identified. The utility function shows the consent of each agent, against the strategy adopted by its opponent. In the model presented in this paper, the factors that seem to affect network formation are used as parameters of the utility function. The utility function will be described in the next sections.

In this paper, a random network is used as the basic network of the model. The main model is formed upon this basic network. To form the main model in this paper, instead of the Nash equilibrium, the concepts of pairwise stability is used. In the next parts of this section, the concept of pairwise stability and the basic networks will be described in detail.

### 3.2. Pairwise Stability

Conceptually, in light of strategic network formation, forming a link is usually a pairwise decision. This means in forming a link, both sides of the relationships tend to form the link while leaving the relation is a one-way decision (Calvó-Armengol and İlkılıç, 2009).

To model this concept, something stronger is needed than Nash-equilibrium in non-cooperative solutions in game theory. In that, the concept of pairwise stability is used in this paper. Assume that *i, j* are two nodes in a network and *u*_*i*_ shows the utility of the node i. Then Network *g* is considered pairwise stable if:

- for all *i, j* ∈ *g, u*_*i*_(*g*) ≥ *u*_*i*_(*g* − *i, j*) and *u*_*j*_(*g*) ≥ *u*_*j*_(*g* − *i, j*), and
- for all *i, j* ∉ *g*, if *u*_*i*_(*g* + *i, j*) > *u*_*i*_(*g*) then *u*_*j*_(*g* + *i, j*) < *u*_*j*_(*g*)

In other words, network g is pairwise stable, if no agent tends to delete a link and no pair of agents tend to make a new link in the network g (Calvó-Armengol and İlkılıç, 2009).

### 3.3. Basic Network of the Model

Most of game-theoretical network formation models have a random network as their basic network. To choose the best random network for the basic network of the presented model, a range of directed and undirected random networks are compared with *C. elegans* frontal neural network using complex network measures. In this section, these random networks and how they are generated for *C. elegans* frontal neural network is described.

#### 3.3.1. Undirected Random Networks

- Watts-Strogatz Random Network: To generate Watts-Strogatz random network, at first, a ring of N nodes is formed where each node connected to *k* nearest neighbors in the ring. This forms a regular network. Assume *u*, *v*, and *w* are three different nodes in the network. Then each edge (*u, v*) would be switched with a new edge (*u, w*) with probability of *P*. Node *w* would be chosen with normal distributed probability from existing nodes (Watts and Strogatz, 1998). In order to generate this network for *C. elegans* frontal neural network, neurons put together in a ring and then each neuron is connected to 5 other neurons based on physical distance. After forming a regular network each edge would be chosen with the probability of 0.5 with another edge. Since edges are more important, probabilities are chosen in a way to make random network proportional to edges of *C. elegans* frontal neural network.
- Expected Degree Random Network: In this random network, an edge is formed between node *u* and node *v* with a probability based on a specific degree distribution. In this paper, undirected *C. elegans* frontal neural network degree distribution is used to make expected degree random network (Newman et al., 2001).
- Power-law Clustering Random network: This random network begins with an empty network and in each time step, a new node is added to the network. In this random network, number of nodes, number of random edges for each randomly added node and probability of adding a new triangle (Cluster) to the network according to randomly added edge is identified (Barabási and Albert, 1999). To generate this random network for *C. elegans* frontal neural network, 131 nodes added to the network. The number of random edges for each added node and the probability of adding a new cluster to the model considered 6 and 0.2, respectively. These parameters are chosen to make random network edges proportional to edges of *C. elegans* frontal neural network.

#### 3.3.2. Directed Random Networks

- Havel-Hakimi Random Network: Havel-Hakimi Random Network is generated based on an in-degree and out-degree sequences. This Network is generated based on the Havel theory (Erdos et al., 2010). To make a directed Havel-Hakimi random network proportional to *C. elegans* frontal neural network the in-degree and the out-degree of *C. elegans* neural network are used. Nodes are chosen with a normal probability distribution.
- Scale-free Random Network: In scale-free random network generation process, edges are added randomly in discrete times. In each time step, a new node might be added to the network. In generation process, α, β, λ, σ_*in*_, and σ_*out*_ are defined as parameters of the model, where α + β + λ = 1, σ_*in*_ and σ_*out*_ are the bias of choosing a new node based on in-degree and out-degree sequences. Note that in this random network model number of nodes is not guaranteed (Barabási et al., 1999). To make a random network proportional to *C. elegans* neural network, the degree distribution of *C. elegans* neural network is used and the probability of adding a new node and a new edge between existing nodes, σ_*in*_ and σ_*out*_ are set to 0.15, 0.8, 0.2 and 0, respectively.
- Erdos-Renyi Random Network: Suppose *N* = {*n*_1_, *n*_2_, …, *n*_*n*_} is set of the nodes. So there could be $\frac{N\left(N-1\right)}{2}$ edges possible between these nodes. A random network can be generated choosing a subset of these edges (Csardi and Nepusz, 2006). To generate a network proportional to *C. elegans* frontal neural network, 131 nodes considered and to generate edge (*u, v*), probability of generating a link between *u* and *v* is set to 0.5.

After generating all directed and undirected networks they compared with *C. elegans* neural network. Between these networks directed Havel-Hakimi random network seem to have more similarities with the *C. elegans* neural network according to measures used for comparison. So, Havel-Hakimi random network is used as the basic network in this paper. Comparisons of these random networks are described in detail in the discussion section.

### 3.4. Utility Function

After defining agents and their strategies, a proper utility function should be defined for the model. To achieve this end, in this paper, several functional and structural properties that seem to have impact on *C. elegans* frontal neural network formation are used as parameters of utility function. These factors include physical distance between the neurons, difference between neurons birth-time, average shortest path of each neuron to the other neurons in the network, page-rank, betweenness centrality, and closeness centrality. Consider *u*_*i*↔*j*_(*g*) is the utility function for neuron *i* in the network *g*, against its opponent that is neuron *j*, *D*_*ij*_ is the physical distance between neuron *i* and neuron *j*, *B*_*ij*_ is the difference between birth-time of neuron *i* and neuron *j*, *S*_*ig*_ is the average shortest path of neuron *i* in the network *g*, *p*_*i*_ is the page-rank of neuron *i* in the network *g*, *C*_*i*_ is the closeness centrality of neuron *i* and finally *Be*_*i*_ is the betweenness centrality of neuron *i* and α, β, λ, θ, ρ and ω are coefficients of the used parameters. Then The utility function for each neuron is made as follow:

(1) $$\begin{array}{l} \left(u_{i↔j}-u_{i↔j}^{\prime }\right)=\alpha \left(D_{ij}\right)+\beta \left(B_{ij}-B_{ij}^{\prime }\right)+\lambda \left(S_{ij}-S_{ij}^{\prime }\right)\\ \;+\rho (P_{i}-P_{i}^{\prime })+\theta (C_{i}-C_{i}^{\prime })+\omega \left(Be_{i}-Be_{i}{}^{\prime }\right)\leq 0 \end{array}$$

## 4. Implementation

To implement the model, first, the coefficients of the utility function should be identified. These coefficients are calculated applying the concept of pairwise stability and using linear programming.

The Pulp python package is used for linear programming (Mitchell et al., 2011). To calculate the limiting constraints for linear programming, with this assumption that the *C. elegans* frontal neural network is pairwise stable, first the utility function is calculated for each pair of neurons with a link between them. Then, the link deleted and again the utility function is calculated in this situation, and the different of these two utilities is used as constraints of linear programming to satisfy the requirements of pairwise stability. The constraints are calculated as depicted in Equation (2).

(2) $$\begin{array}{l} \left(u_{i↔j}-{u^{\prime }}_{i↔j}\right)=\alpha \left(D_{ij}\right)+\beta \left(B_{ij}-{B^{\prime }}_{ij}\right)+\lambda \left(S_{ij}-{S^{\prime }}_{ij}\right)\\ \;+\rho \left(P_{i}-{P^{\prime }}_{i}\right)+\theta \left(C_{i}-{C^{\prime }}_{i}\right)+\omega \left(Be_{i}-Be_{i}{}^{\prime }\right)\leq 0 \end{array}$$

Again for each pair of neurons departed from each other, the utility is calculated in both situations, with and without the link between the neurons, and the difference is used as the constraints. This time, the constraints are calculated using Equation (3).

(3) $$\begin{array}{l} \left(u_{i↔j}-u_{i↔j}{}^{\prime }\right)=\alpha \left(D_{ij}\right)+\beta \left(B_{ij}-B_{ij}{}^{\prime }\right)+\lambda \left(S_{ij}-S_{ij}{}^{\prime }\right)\\ \;+\rho \left(P_{i}-P_{i}{}^{\prime }\right)+\theta \left(C_{i}-C_{i}{}^{\prime }\right)+\omega \left(Be_{i}-Be_{i}{}^{\prime }\right)\geq 0 \end{array}$$

These constraints are calculated for each pair of neurons and added to the dictionary of the constraints. Dictionary of constraints is a data structure that is used to pass the constraints to the Pulp for linear programming.

In the next step, an objective function should be identified to minimize by linear programming. Thus, the sum of all utilities for all the neurons is used as the objective function of linear programming. Then, linear programming is applied with the defined constraint dictionary. The objective function and the required coefficients are calculated, accordingly. The coefficients after applying linear programming are shown in Table 1. Pulp uses heuristic algorithms to solve linear programming problems.

**Table 1:** Calculated coefficients for utility function.

**Parameters**	**α**	**β**	**λ**	**ρ**	**θ**	**ω**
Calculated coefficient	−1	−1	−0.02344	0	−1	0

Since the problem space is not convex, it doesn't guarantee the optimum answer and may stop in local optimums. Note that only satisfying constraints is critical and suffice here and the global minimum solution is not sought for.

The result shows that throughout all factors assumed to have the impact on the formation of *C. elegans* frontal neural network, physical distance, the difference of birth times, average shortest path, and closeness centrality affect the *C. elegans* neural network formation. The negative sign in the result also shows that in the formation of *C. elegans* neural network, neurons try to shorten their physical and functional distance, have lower closeness centrality and make links with neurons with closest birth time. After determining the utility function, the final strategic model for *C. elegans* network formation can be created.

The utility function should be applied on directed Havel-Hakimi random network that was chosen as the basic network of the model. To use physical distance and birth time as the parameters, Havel-Hakimi nodes should be mapped to the real neurons in *C. elegans* frontal neural network. To do this, in-degree and out-degree for each node are added together in both directed Havel-Hakimi and *C. elegans* frontal neural network and then sorted ascendingly. Thus, two sorted list of *D*_*h*_ = {*D*_*h*1_, *D*_*h*2_, …, *D*_*hn*_} and *D*_*c*_ = {*D*_*c*1_, *D*_*c*2_, …*D*_*cn*_} are created, in which *D*_*hn*_ stands for the sum of the in-degree and out-degree of node *n* in the Havel-Hakimi random network, and *D*_*cn*_ denotes the sum of in-degree and out-degree of node *n* in the *C. elegans* neural network. Now, the nodes can be mapped to neurons based on these sorted lists. For instance, a node with degree *D*_*h*1_ would be mapped to a neuron with degree *D*_*c*1_. To form the final model, again pairwise stability is used as follow:

- For each pair of nodes connected to each other, the utility of each node is calculated using the utility function. Then, the link between them is deleted and the new utility is calculated again for both nodes. If the utility of each node increases, the link is deleted from Havel-Hakimi random network, otherwise, the link will be kept.
- For each pair of departed nodes in Havel-Hakimi random network, the utility of the nodes is calculated using utility function. Then, a new link is added between these nodes and the utility is calculated again for each node in this state. If the utility increases for both nodes, a new link will be added to Havel-Hakimi random network, otherwise, the state of the network will remain unchanged.

**Figure 1:**
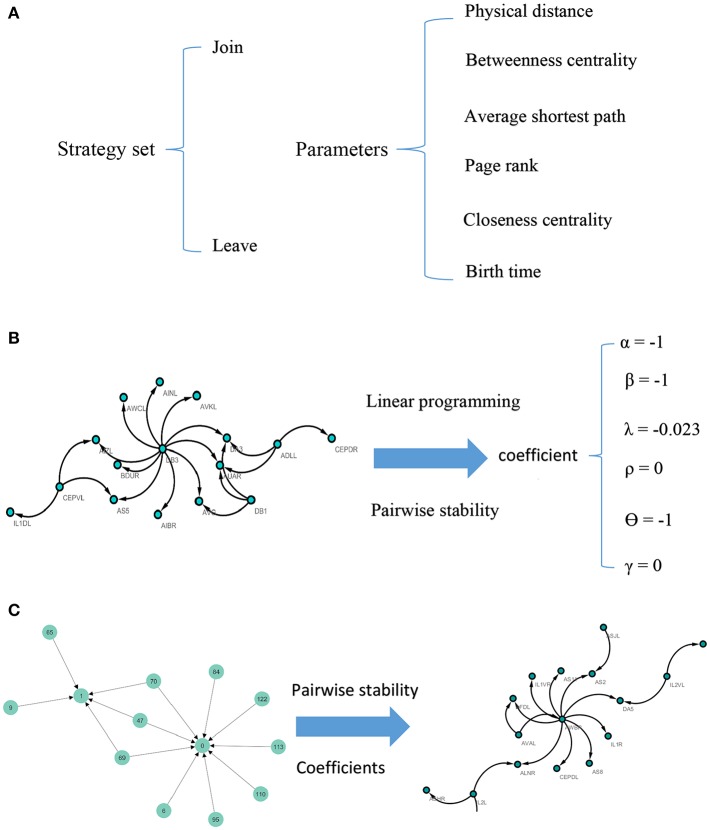
Visual illustration of the model. For each pair of nodes, the strategy set encompasses two actions. The potential factors affect the *C. elegans* neural network are used as parameters of the model **(A)**. To calculate the coefficients of the parameters, linear programming and concept of pairwise stability are applied to the *C. elegans* frontal neural network **(B)**. The game-theoretical model is made upon Havel-Hakimi random network using coefficients calculated in previous steps. The nodes are considered as agents and the game is continued until the conditions for pairwise stability are met **(C)**.

Note that according to the concept of pairwise stability, adding a new link is a pairwise decision. However, deleting a link is the decision that each agent in the relation makes by itself. A concise description of the model can be seen in Figure 1.

## 5. Discussion

In this section, first, the choice of the best random network for the basic network is evaluated and described. Then, the strategic model is built upon the chosen basic network. Finally, the proposed model will be compared with random networks and previous models in the literature for C. elegans neural network formation.

### 5.1. Evaluation of the Random Networks

As previously mentioned, directed Havel-Hakimi random network was chosen as the basic network of the mode. In this part, the logic behind this selection will be described by evaluation of different random networks.

#### 5.1.1. Evaluation of Undirected Random Networks

One can see neural networks as undirected graphs for simplicity. In this paper, some undirected networks are compared with *C. elegans* frontal neural network to assess the performance and availability of this assumption.

In Table 2, average clustering coefficient, average shortest path, average neighbors, network diameter, betweenness centrality, network homogeneity, network density for Watts-Strogatz, expected degree distribution, and power-law clustering random networks are presented. As it can be seen in Table 2, clustering coefficient, the average of neighbors, networks density of Watts-Strogatz, and expected degree of random networks are very different with the ones of *C. elegans* frontal neural network.

**Table 2:** Comparison between undirected random networks.

	**Average clustering coefficient**	**Average shortest path length**	**Average neighbors**	**Network diameters**	**Betweenness**	**Network homogeneity**	**Network density**
*C. elegans* frontal neural network	0.24	2.523	10.48	6	0.160226	0.54463	0.0806
Watts Strogatz network	0.08	3.807	4.0	7	0.015623	0.29628	0.0307
Expected degree network	0.08	2.672	6.42	6	0.160585	0.68080	0.0648
Power-law clustering network	0.23	2.208	11.28	4	0.318067	0.72531	0.0867

**Figure 2:**
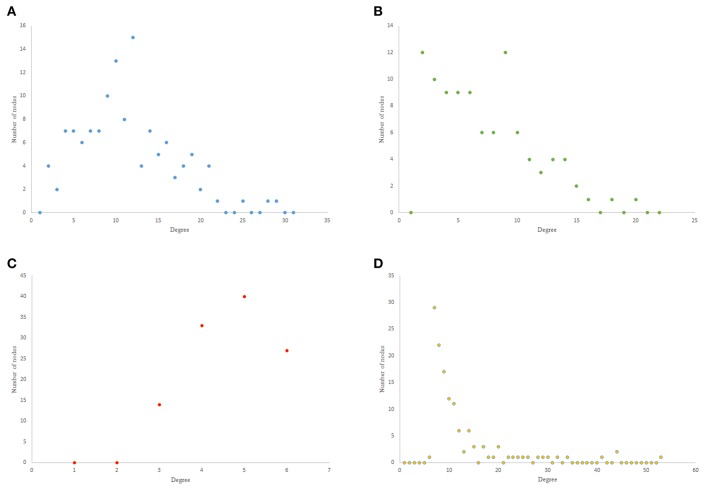
Undirected networks degree distribution. **(A)** *C. elegans* degree distribution. **(B)** Expected degree distribution random network degree distribution. **(C)** Watts-Strogats random network degree distribution. **(D)** Power-low clustering random network degree distribution.

The results from power-law clustering random network and the ones in *C. elegans* frontal neural network, specially for the case of clustering coefficient, have more similarities compared to the other models. The most significant difference between *C. elegans* frontal neural network and power-law clustering is in network diameter and network homogeneity. Among undirected random networks, power-law clustering seems to be the most similar random network to the *C. elegans* frontal neural network. This resemblance can be the result of *C. elegans* power-law degree distribution. In Figures 2A–D, the degree distribution of the *C. elegans* frontal neural network and these random networks are shown.

Figure 2A depicts the degree distribution for undirected *C. elegans* frontal neural network. As it can be seen, the majority of nodes have a degree between 10 and 40. However, the degree distributions for expected degree distribution network and Watts-Strogatz random network, on the contrary, show a distribution between 0 and 10 for most of the nodes. As Figures 2B,C suggest, the degree distribution of The expected degree distribution random network and the Watts-Strogats random network degree distributions are very different from the degree distribution of the *C. elegans* frontal neural network.

As Figure 2D suggests, power-law clustering random network degree distribution, similar to undirected *C. elegans* frontal neural network, shows a distribution between 10 and 40 for predominant of the nodes.

#### 5.1.2. Evaluation of Directed Random Networks

On the other hand, the interactions between neurons through chemical synapses is directional. With this in mind, some directed random networks are also compared with *C. elegans* frontal neural network. In Table 3, measurements of average clustering coefficient, average shortest path length, average neighbors, and network diameter for directed Havel-Hakimi, Erdos-Renyie, and the scale-free random networks are shown.

**Table 3:** Comparison between directed random networks.

	**Average clustering coefficient**	**Average shortest path length**	**Average neighbors**	**Network diameter**
*C. elegans* frontal neural network	0.24	3.12	10.48	9
Directed Havel-Hakimi random network	0.12	1.98	11.72	6
Erdos-Renyie random network	0.56	2.42	13.28	5
Scale-free random network	0.16	2.04	3.49	5

For all of these three directed random networks, the average shortest path length is nearly similar to the one for *C. elegans* frontal neural network. Although the average clustering coefficient of Erdos-Renyie is very different from the average clustering coefficient of *C. elegans* neural network, the predicted values for this coefficient are very similar between *C. elegans* frontal neural network, Havel-Hakimi, and scale-free network. The average number of neighbors and network density are also similar between *C. elegans* frontal neural network, Havel-Hakimi and the random scale-free network.

**Figure 3:**
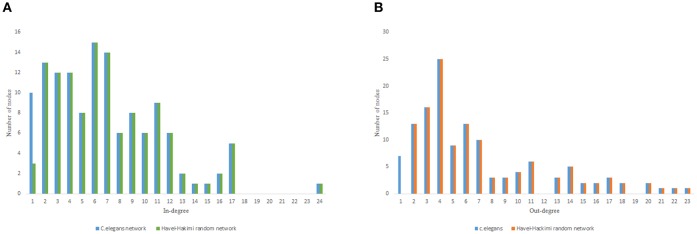
*C. elegans* frontal neural network and Havel-Hakimi random network degree distribution. **(A)** *C. elegans* and Havel-Hakimi random network in-degree distribution. **(B)** *C. elegans* and Havel-Hakimi random network out-degree distribution.

**Figure 4:**
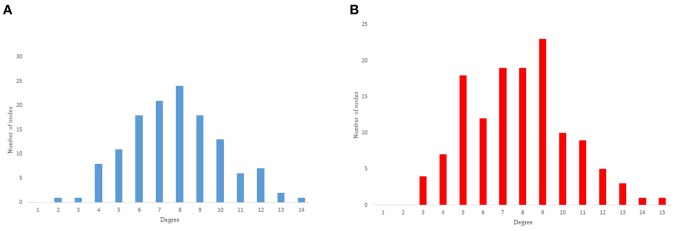
Erdos-Renyie degree distribution. **(A)** Erdos-Reynie in-degree distribution. **(B)** Erdos-Reynie out-degree distribution.

**Figure 5:**
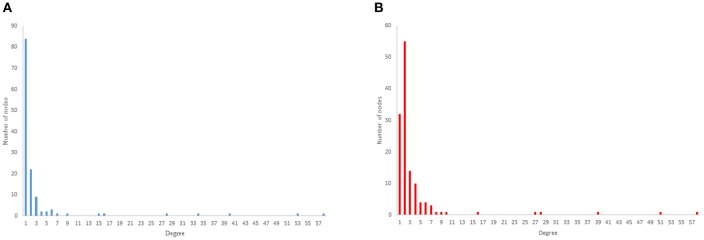
Scale-free degree distribution. **(A)** Scale-free in-degree distribution. **(B)** Scale-free out-degree distribution.

As previous studies indicate, *C. elegans* neural network has some characteristics of scale-free networks. The resemblance between *C. elegans*, power law clustering random network and the random scale-free network can be the result of power-law degree distribution in *C. elegans* neural network. In Figures 3–5 the histograms of degree distribution of *C. elegans* neural network and these directed random networks are shown.

Figure 3 depicts the degree distribution for *C. elegans* frontal neural network and the Havel-Hakimi random network. As it can be seen, the in-degree distribution for predominant of the nodes is between 1 and 15 and the majority of the nodes have an out-degree distribution between 1 and 25 for both the *C. elegans* and the Havel-Hakimi random network. Since the Havel-Hakimi random network is created based on the *C. elegans* in-degree and out-degree distribution and it tries to retain the degree distribution, it is no surprise that the in-degree and out-degree distribution of these two networks is so similar to each other. However, the degree distribution is used as a factor to compare networks, for the degree distribution may be coded in DNA of the worm somehow.

**Figure 6:**
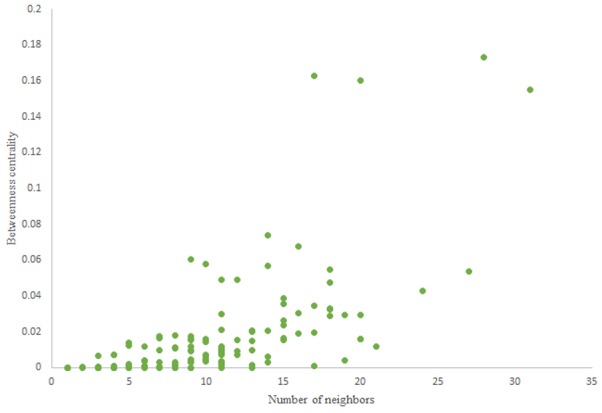
Betweenness centrality of *C. elegans* frontal neural network.

**Figure 7:**
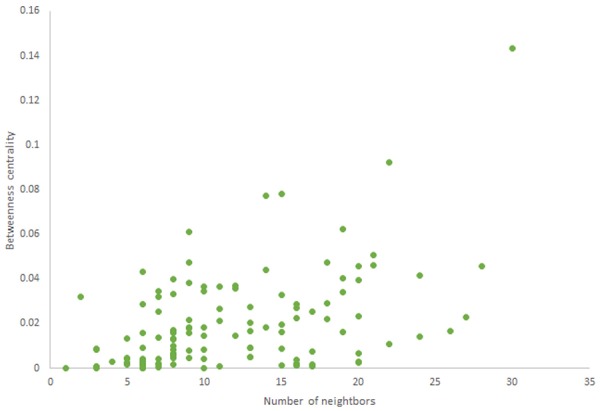
Betweenness centrality of Havel-Hakimi random network.

**Figure 8:**
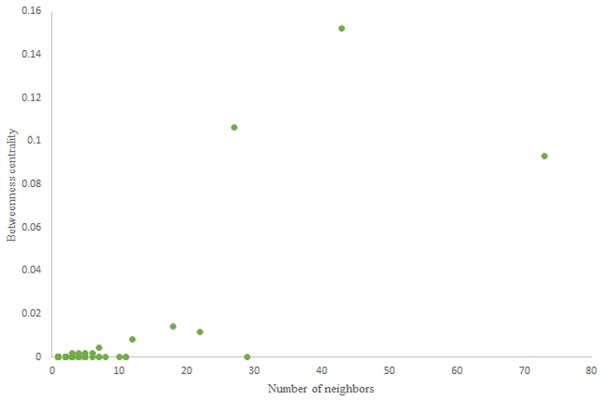
Betweenness centrality of scale-free random network.

**Figure 9:**
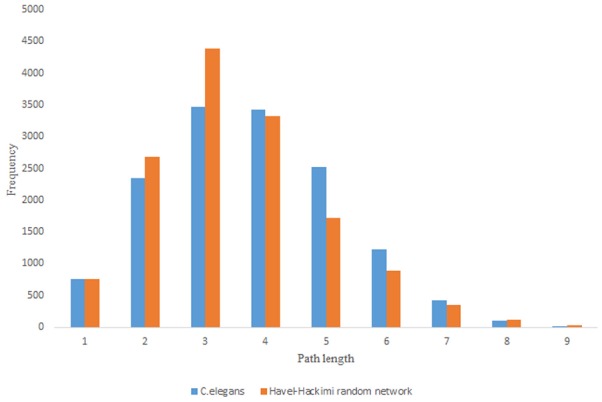
*C. elegans* frontal neural network and Havel-Hakimi random network shortest path distribution.

**Figure 10:**
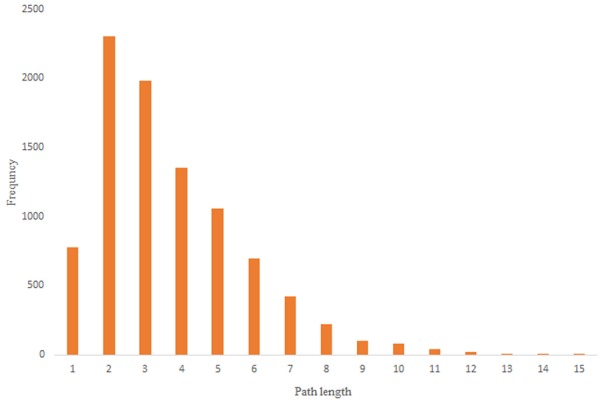
Scale-free random network shortest path distribution.

For more comparison between Havel-Hakimi and other directed random networks, the betweenness centrality and the shortest path distribution of *C. elegans* and directed random networks are depicted in Figures 6–10. As Figure 9 suggests the shortest path distribution of the *C. elegans* frontal neural network and The Havel-Hakimi random network are also more similar to each other in comparison with other directed random networks. According to all aspects of directed and undirected networks, directed Havel-Hakimi random network was chosen as the basic network of the model.

### 5.2. Evaluation of the Main Model

After choosing the basic network for the model, using pairwise stability concept and utility function described in the previous section, the game-theoretical model is created. The output of the model is a network.

According to the development process of directed Havel-Hakimi random network, the nodes with higher degrees join together in the first place. Based on the resemblance between directed Havel-Hakimi random network and *C. elegans* frontal neural network, it can be concluded that in the formation of *C. elegans* frontal neural network, or maybe all neural networks in general, hubs may play a critical role. The process of creating the model is described in the implementation section. In Table 4 the network resulted from the game-theoretical model is compared with *C. elegans* frontal neural network and directed Havel-Hakimi random network. In this table, the clustering coefficient, average shortest path, average neighbors, and network diameter are compared. As Table 4 suggests, the difference between average shortest path length of the game-theoretical model and the *C. elegans* frontal neural network is 0.98, which is closer compared to the directed Havel-Hakimi random network with a difference of 1.14. The average neighbors of the game-theoretical model are also more similar to the *C. elegans* neural network with a difference of 0.66 in comparison with the directed Havel-Hakimi random network which has a difference of 1.24. As it can be seen, the network diameter of the strategic model is improved by 1 compared to the Havel-Hackimi random network. As Table 4 depicts, in the comparison between network forms from the game-theoretical model and directed Havel-Hakimi random network, which is the basic network of the model, all measures improved and became more similar to the *C. elegans* frontal neural network, except for betweenness centrality that is worsened by 0.002.

**Table 4:** Comparison between *C. elegans* and proposed model.

	**Average clustering coefficient**	**Average shortest path length**	**Average neighbors**	**Network diameter**
*C. elegans* frontal neural network	0.24	3.12	10.48	9
Directed Havel-Hakimi random network	0.12	1.98	11.72	6
Strategic model for *C. elegans* f.n.n.	0.118	2.14	11.14	7

**Figure 11:**
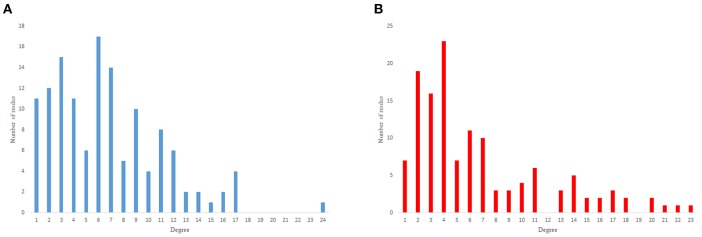
Game-theoretical network formation model degree distribution. **(A)** Game theoretical model in-degree distribution. **(B)** Game theoretical model out-degree distribution.

**Figure 12:**
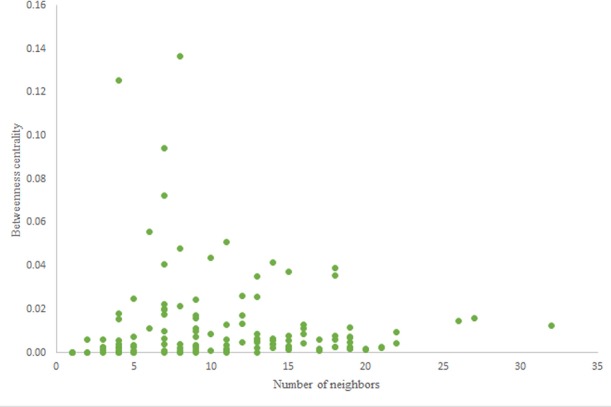
Game-theoretical model betweenness centrality distribution.

**Figure 13:**
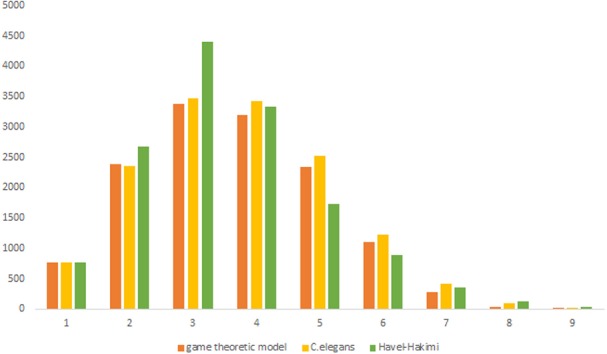
Game-theoretical model shortest path length.

The in-degree and out-degree distributions, shortest path length distribution, and betweenness centrality distribution of the network generated using game theory are shown in Figures 11–13. As Figure 12 illustrates, predominant of the nodes that have 1–22 neighbors have a betweenness centrality between 0 and 0.04. As previously depicted in Figures 6, 7. The *C. elegans* have a betweenness centrality between 0 and 0.04 for nodes that have between 1 and 22 neighbors and the Havel-Hakimi random network have a betweenness centrality between 0 and 0.06 for nodes that have between 1 and 22 neighbors. In Figure 13 the shortest path distribution of the game-theoretical model is compared with the *C. elegans* frontal neural network and the Havel-Hakimi random network. As the figure suggests, the game-theoretical model is more similar to the *C. elegans* frontal neural network in comparison with the Havel-Hakimi random network. In that, from these figures and results that are reported in Table 4, it seems that the game theoric model has more in common with the *C. elegans* neural network compared to the Havel-Hakimi random network.

The game-theoretical model for *C. elegans* neural network formation is also compared with the regular network and the distance based random model that was proposed by Itzhack and Louzoun in Table 5. In order to compare the new proposed model with distance based random model, this model is also implemented for the *C. elegans* frontal neural network. As illustrated in Table 5, game-theoretical model leads to the most similar network with *C. elegans* frontal neural network.

**Table 5:** Comparison between game-theoretical model and previously proposed models.

	**Average clustering coefficient**	**Average shortest path length**	**Average neighbors**	**Network diameter**
*C. elegans* frontal neural network	0.24	3.12	10.48	9
Game-theoretical model	0.118	2.14	11.14	7
Regular network	0.77	8.11	11.61	6
Distance based random model	0.48	1.46	86.10	3

In the networks compared in Table 5, regular network model predicts average neighbors and network density better than the other models except for game-theoretical model. But it fails to predict average clustering coefficient and average shortest path length of *C. elegans* frontal neural network. On the other hand, although distance based random predicts the clustering coefficient and the average shortest path better than regular network model, it fails in the other aspects. This shows neural networks are somewhere in between random and regular networks as neither of these models can explain neural network formation by itself.

As previously stated in the methods section, various structural properties include physical distance, birth time, and different functional properties, such as betweenness centrality, page rank, average shortest path as well as closeness centrality are used to implement the game-theoretical model. the results suggest that between all these factors, physical distance, birth time, average shortest path, and betweenness centrality have more influence on the formation of the *C. elegans* frontal neural network. According to the results, game theoric model suggested here explain the formation of the *C. elegans* neural network more accurately compared to previously suggested models. It also takes more properties into account. Since the game theoric network uses the Havel-Hakimi random network as the basic network, according to the process of creating the Havel-Hakimi model, it highlights some important features, such as the importance of the hubs in nervous systems.

It worth mentioning that using genetic data and using more complex equation instead of a simple linear relation between factors in utility function may result in a more accurate model. Furthermore, as mentioned in the introduction section, stochastic resonance and the heterogeneity of synaptic input connectivity can also be translated to factors that can be used in the utility function. While using these parameters can lead to more accurate and more descriptive model, adding these features to the model would increase the complexity of the model exponentially. In the future, using more computational resources, these features can be added to this model and more complex relationship between factors in the utility function can be used to make a more accurate and descriptive model.

## 6. Conclusion

Understanding how the nervous system is formed and how the structure of the neural network affect the functional properties of the nervous system can shed light on the understanding of diseases like dementia and autism.

In this paper, a new model based on game theory is proposed for the formation of *C. elegans* frontal neural network. In the model introduced in this work, neurons are considered as agents of the system that try to maximize their benefits posing their best strategies. The strategy set for each neuron includes making a link or removing an existing link with other neurons in the network. Neurons are assumed to be self-incentive and the model is implemented as a non-cooperative game-theoretical approach.To create the model, directed Havel-Hakimi random network is chosen for the basic network of the model. The main model is built on this random network. After choosing the best basic network, utility function is defined and potentially influential factors are used as parameters of the utility function. To find these factors coefficient, linear programming is used. Then, by the use of pairwise stability concept, the main model is constructed upon the basic network and using utility function. The results show that the presented model can describe the formation of *C. elegans* frontal neural network better than random and regular networks and previously proposed models. Based on this model, it can be concluded that physical distance, the difference of neurons birth-time, average shortest length, and the closeness centrality can affect the formation of neural networks with this assumption that all neural networks have some fundamental formation processes in common.

In future works, the focus would be on more complex neural networks like mammalian and homo sapiens neural networks and on simulation of the complex neural networks on the computer based on formation processes. The simulators that use formation processes and factors impacting the neural network formation may create neural networks more accurately. They also can be used to simulate the evolution of the neural networks, as well.

## Data Availability

Publicly available datasets were analyzed in this study. This data can be found here: http://www.wormatlas.org/.

## Author Contributions

MK conceived of the presented idea, developed the theory, performed the computations, and wrote the manuscript. SG supervised the project, helped with the development of the idea, and reviewed the manuscript. HV helped with supervising the project, helped with the development of the idea, and reviewed the manuscript.

### Conflict of Interest Statement

The authors declare that the research was conducted in the absence of any commercial or financial relationships that could be construed as a potential conflict of interest.
